# A Specific Signature of Circulating Free Fatty Acid Discriminates Bullous Pemphigoid From Pemphigus Vulgaris and Healthy Controls

**DOI:** 10.1111/exd.70228

**Published:** 2026-02-20

**Authors:** Simone Baldi, Sara Bertorello, Francesco Cei, Giulia Nannini, Marta Menicatti, Elena Niccolai, Gianluca Bartolucci, Maria Efenesia Baffa, Carlo Pipitò, Emiliano Antiga, Amedeo Amedei, Roberto Maglie

**Affiliations:** ^1^ Department of Experimental and Clinical Medicine University of Florence Florence Italy; ^2^ Department of Neuroscience, Psychology, Drug Research and Child Health NEUROFARBA University of Florence Florence Italy; ^3^ Department of Health Sciences, Section of Dermatology University of Florence Florence Italy

**Keywords:** bullous pemphigoid, free fatty acids, short chain fatty acids, zonulin

## Abstract

Bullous pemphigoid (BP) is an autoimmune skin disorder marked by antibodies targeting basement membrane proteins BP180 and BP230. Recent evidence suggests a role for the gut–skin axis and microbial metabolites, especially short‐chain fatty acids (SCFAs), in modulating skin homeostasis and immune responses. In this study, we investigated the gut permeability and evaluated the circulating free fatty acids (FFAs) in BP patients, along with the assessment of the ability of each FFA to discriminate BP patients from both pemphigus vulgaris (PV) patients and healthy controls (HC). Thirty‐six BP patients and 36 sex‐ and age‐matched HC were enrolled. In addition, we used a previously examined cohort of 18 PV patients. FFAs were quantified through gas chromatography–mass spectrometry. Serum zonulin levels were measured by ELISA test and then correlated with FFA levels and clinical markers of disease activity. Receiver operating characteristic (ROC) curve analyses evaluated the diagnostic utility of individual FFAs. BP patients had significantly lower SCFA levels but higher medium‐chain (MCFAs) and long‐chain fatty acids (LCFAs) than HC. Zonulin levels were elevated in BP and correlated negatively with isovaleric acid. No clear associations emerged between FFAs, zonulin and clinical disease severity. Sparse partial least square discriminant analysis identified propionic, octanoic and octadecanoic acids as key discriminators between BP and both HC and PV serum FFAs. These metabolites achieved ROC AUCs > 0.9, showing a strong diagnostic value. Our findings reveal a pro‐inflammatory shift in serum FFA profiles in BP—marked by decreased SCFAs and increased MCFAs/LCFAs—concurrent with elevated gut permeability. The strong diagnostic performance of propionic, octanoic and octadecanoic acids highlights their promise as biomarkers for BP.

## 1 Introduction

Bullous pemphigoid (BP) is the most common autoimmune bullous disease, predominantly affecting elderly people [1]. It is characterised by the presence of autoantibodies targeting basement membrane zone (BMZ) antigens, primarily the hemidesmosome proteins BP180 and BP230. These autoantibodies trigger an inflammatory cascade involving complement activation and recruitment of myeloid granulocytes to the skin, lastly resulting in dermal‐epidermal separation [2]. Clinically, BP presents with tense blisters and intense pruritus, and its management is often complicated by the advanced age of patients and associated comorbidities, such as cardiovascular diseases and diabetes, which significantly affect prognosis [1]. Pathogenically, BP is an autoantibody‐mediated disease driven by type 2 (T2) immunity and therapies targeting T2‐associated cytokines, mainly interleukin‐(IL)4 and IL‐13, have shown remarkable clinical efficacy in patients with steroid‐resistant or recalcitrant disease [3, 4].

The host microbiota has recently been identified as a crucial factor influencing skin health, contributing to skin homeostasis, protection against pathogens and immune regulation [5]. In detail, studies indicate that the microbiota composition of lesional skin in conditions like atopic dermatitis and psoriatic disease differs significantly from that of healthy skin [6, 7]. Similarly, studies have highlighted the critical role of skin microbiota in the BP pathogenesis [8].

Emerging evidence also underscores the significance of the gut–skin axis, highlighting the role of the gut microbiome (GM) in skin health and disease [9]. Notably, many patients with inflammatory bowel disease (IBD) suffer from dermatological manifestations, and several inflammatory skin disorders are associated with gut dysbiosis characterised by altered GM diversity and composition [10]. A recent study by Liu and colleagues documented that BP patients exhibit a diminished intestinal microbial diversity, with increased abundance of *Flavonifractor* spp. and a decreased level of *Faecalibacterium* spp., a bacterial genus known for its anti‐inflammatory properties [11].

This complex and bidirectional gut–skin axis is thought to involve microbial metabolites, such as short‐chain fatty acids (SCFAs), which play critical roles in enhancing epithelial barrier function and modulating the immune system [12]. Interestingly, butyric acid‐producing bacteria have been shown to be significantly reduced in pemphigus patients compared to healthy individuals [12]. Consistent with these findings, our recent study identified a marked reduction in SCFA levels in patients with pemphigus vulgaris (PV) compared to healthy people [13]. Based on these observations, we hypothesized that BP might similarly exhibit an imbalance in circulating free fatty acids (FFAs), potentially driven by gut dysbiosis. To test this hypothesis, we analysed gut permeability and evaluated serum FFA concentrations in a retrospective cohort of untreated BP patients. Additionally, we investigated potential associations between FFA profiles, disease severity and markers of gut permeability. Finally, we assessed the potential discriminatory ability of FFAs in distinguishing BP patients from healthy and disease (PV patients) controls.

## 2 Materials and Methods

### 2.1 Patients

We retrospectively analysed serum samples from 36 patients diagnosed with BP who were followed at the Dermatology Section of the University of Florence (Italy). For comparison, 36 sex‐ and age‐matched healthy subjects were included as controls (HC). Additionally, we compared these BP samples with serum data from a previously examined cohort of 18 PV patients (disease controls) [13]. BP diagnosis was confirmed according to the following criteria: (i) presence of subepidermal blistering identified via histopathology; (ii) linear IgG/C3 deposition along the BMZ observed in perilesional skin by direct immunofluorescence; (iii) detection of circulating anti‐BMZ antibodies using indirect immunofluorescence; and (iv) quantification of serum anti‐BP180/BP230 IgG antibodies through Enzyme Linked Immunosorbent Assay (ELISA). All serum samples were collected at the time of enrolment, prior to the treatment starting. Patients who met any of the following exclusion criteria were not included in the study: (i) a documented history of chronic gastroenteric disorders or malabsorption; (ii) use of antibiotics, pre/pro/synbiotics or postbiotics within 8 weeks before enrolment; and (iii) treatment with prednisone at a dose ≥ 0.3 mg/kg/day or steroid‐sparing immunosuppressants within 4 weeks prior to enrolment. Demographic and clinical data, including sex, age, comorbidities and disease severity as assessed by the Bullous Pemphigoid Disease Activity Index (BPDAI), are summarised in Table 1.

**TABLE 1:** Clinical and immunopathological characteristics of patients with bullous pemphigoid enrolled in the study.

Characteristics	BP, *N* = 36
Age at diagnosis, years, mean (SD)	76.16 (10.89)
Sex, *n* (%)	
Male	19 (52.8)
Female	17 (47.2)
Comorbidities, *n* (%)	
Cardiovascular disease	20 (76.7)
Neurologic degenerative disease	7 (23.3)
Diabetes mellitus	14 (26.7)
Neoplasm	6 (16.7)
Renal disease	3 (13.3)
BPDAI scores, median [IQR]	56 [25.5–81.50]
BP subtype, *n* (%)	
Bullous	32 (88.8)
Prurigo‐like	3 (8.3)
Urticaria‐like	1 (2.7)

The study was conducted in accordance with the Declaration of Helsinki and was approved by the local Ethics Committee (CEAVC 2023‐08313725). All participants provided written informed consent prior to enrolment.

### 2.2 Evaluation of Serum Free Fatty Acids by GC–MS Analysis

FFAs, namely circulating SCFAs (acetic, propionic, butyric, isobutyric, 2‐methylbutyric, isovaleric and valeric acids), MCFAs (hexanoic, octanoic, decanoic and dodecanoic acids) and LCFAs (tetradecanoic, hexadecanoic and octadecanoic acids), were analysed using an Agilent GC–MS system composed of an HP 5971 single quadrupole mass spectrometer, an HP 5890 gas chromatograph and an HP 7673 autosampler, according to our previously described protocols [14, 15]. Briefly, just before the analysis, each sample was thawed and the FFAs were extracted as follows: an aliquot of 200 μL of serum sample was added to 10 μL of ISTD mixture, 100 μL of tert–butyl methyl ether and 20 μL of 6 M HCl + 0.5 M NaCl solution in a 0.5 mL centrifuge tube. Each tube was then vortexed for 2 min and centrifuged at 10 000 rpm for 5 min, and finally, the solvent layer was transferred to a vial with a microvolume insert for GC–MS analysis.

### 2.3 Serum Zonulin Assay

Serum zonulin levels were measured using the IDK Zonulin ELISA kit (Immundiagnostik AG, Germany) according to the manufacturer's instructions. The assay utilised a competitive ELISA method [16]. First, a biotinylated zonulin tracer was added to the serum samples, which were then transferred to a 96‐well plate pre‐coated with polyclonal anti‐zonulin antibodies. During the incubation period, free zonulin in the samples competed with the biotinylated zonulin tracer for binding to the immobilised antibodies. Unbound components were removed by three successive washing steps. In the subsequent incubation, peroxidase‐labelled streptavidin, which binds to the biotinylated zonulin tracer, was added to the wells. After another three washing steps, a peroxidase substrate was added. Finally, the enzymatic reaction was halted using a stop solution, and absorbance was measured at 450 nm using a photometer. To quantify the zonulin concentration, a dose–response curve of absorbance values (optical density at 450 nm) versus concentration was generated using the kit's standard values. Zonulin levels in both patient and control samples were determined based on this standard curve and expressed in ng/mL.

### 2.4 Statistical Analysis

Statistical analyses were conducted in R 4.2 using the packages stats (v.4.2.2), vegan (v.2.6.2), MixOmics (v.6.20.0), pROC (v.1.18.5) and other packages satisfying their dependencies. Data visualisation and plotting were performed with ggplot2 (v.3.4.0) and ggpubr (v.0.6.0). Differences in FFAs and zonulin levels between BP patients and HC were assessed using the non‐parametric Mann–Whitney test. Spearman's rank correlation was employed to evaluate monotonic relationships between FFA and zonulin levels. Principal coordinates analysis (PCoA) based on Bray–Curtis dissimilarity of proportional abundances was used to assess group differences. These differences were statistically tested using permutation‐based methods, including PERMANOVA and Betadisper, with 9999 permutations.

In analyses involving all three groups (HC, BP and PV), FFAs differences were initially tested using the Kruskal–Wallis test, followed by Dunn's post hoc test when appropriate. However, due to imbalances in age and sex distributions—particularly between BP and PV—potential confounding was addressed in two ways. First, age and sex were included as covariates in the PERMANOVA model for beta diversity, enabling adjustment for these factors in multivariate analysis. Second, as non‐parametric tests like Kruskal–Wallis cannot control for covariates, linear regression models were subsequently applied to FFAs data to assess group differences while adjusting for age and sex. This approach allowed for a more accurate interpretation of potential biomarker variations. Sparse partial least square discriminant analysis (sPLS‐DA) was conducted on standardised quantities using the MixOmics package, with settings optimised for both reproducibility and prediction power. To further assess the discriminatory potential of individual FFAs identified by sPLS‐DA, pairwise receiver operating characteristic (ROC) curve analysis was performed. The area under the curve (AUC) was calculated for each ROC curve to quantify diagnostic performance. The optimal cut‐off value for each FFA was determined using Youden's index, which maximises the combined sensitivity and specificity. Based on these cut‐offs, subjects were classified into BP, HC or PV groups. To evaluate the accuracy of these cut‐offs, contingency tables (confusion matrices) were generated by comparing predicted classifications with the actual group assignments. From these tables, true positive (TP), true negative (TN), false positive (FP) and false negative (FN) counts were calculated. All *p*‐values obtained from multiple comparisons were adjusted using the Benjamini–Hochberg method, with *p* < 0.05 considered statistically significant.

## 3 Results

### 3.1 Evaluation of Serum FFAs in Bullous Pemphigus Patients

**FIGURE 1:**
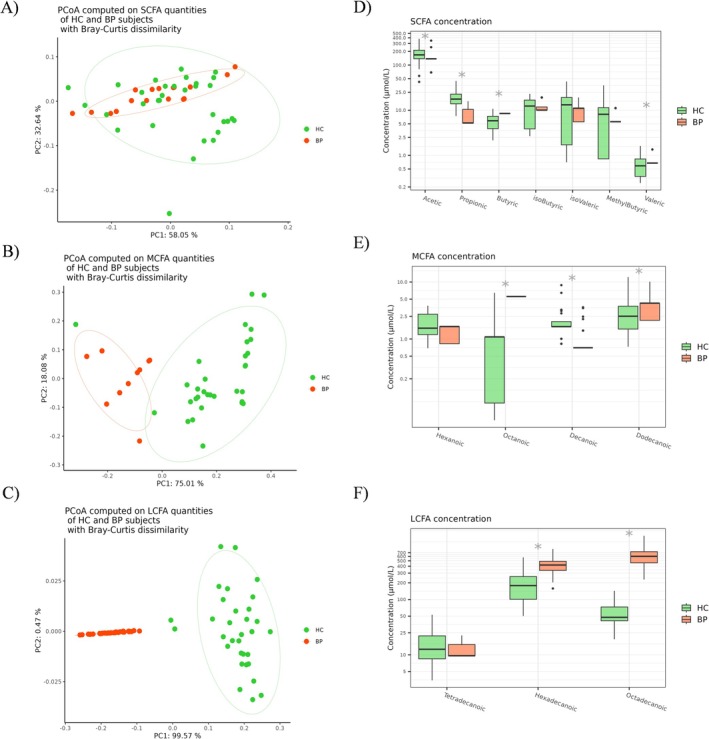
Principal component analysis (PCoA) conducted with the Bray–Curtis dissimilarity index revealed significant differences in the SCFA (A), MCFA (B) and LCFA (C) quantities between sera of BP patients and HC. Statistical significance was assessed using permutational multivariate analysis of variance (PERMANOVA). Box plots representing differences in the serum levels (μmol/L) of SCFAs (D), MCFAs (E) and LCFAs (F) between BP patients and HC. Analyses were assessed using the Mann–Whitney (Benjamini–Hochberg post‐test) and asterisks (*) represent *p* adjusted values < 0.05. BP, bullous pemphigoid; HC, healthy control; LCFA, long‐chain fatty acid; MCFA, medium‐chain fatty acid; SCFA, short‐chain fatty acid.

PCoAs of FFA quantities (μmol/L) (Table S1), computed using the Bray Curtis dissimilarity index, revealed significant differences in SCFA (PERMANOVA < 0.0001), MCFA (PERMANOVA < 0.0001) and LCFA (PERMANOVA < 0.0001) profiles of BP patients compared to HC (Figure 1A–C). Compared to HC, BP patients showed a lower total amount of circulating SCFAs (*p*adj = 0.002) (Figure S1A), while the total levels of MCFAs (*p*adj < 0.0001, Figure S1B) and LCFAs (*p*adj < 0.0001) were higher (Figure S1C). Focusing on single FFAs, BP patients showed lower concentrations of acetic (*p*adj < 0.0001) and propionic (*p*adj < 0.0001) acids, but higher levels of butyric (*p*adj < 0.0001) and valeric (*p*adj = 0.009) acids (Figure 1D) compared to HC. Regarding MCFAs, BP patients had elevated abundances of octanoic (*p*adj < 0.0001) and dodecanoic (*p*adj = 0.031) acids, but a decreased concentration of decanoic acid (*p*adj < 0.0001) compared to HC (Figure 1E). Finally, concerning LCFAs, BP patients showed significant increases in both hexadecanoic (*p*adj < 0.0001) and octadecanoic acid (*p*adj = 0.002) levels compared to HC (Figure 1F).

### 3.2 Serum Zonulin Levels and Correlation With FFAs

**FIGURE 2:**
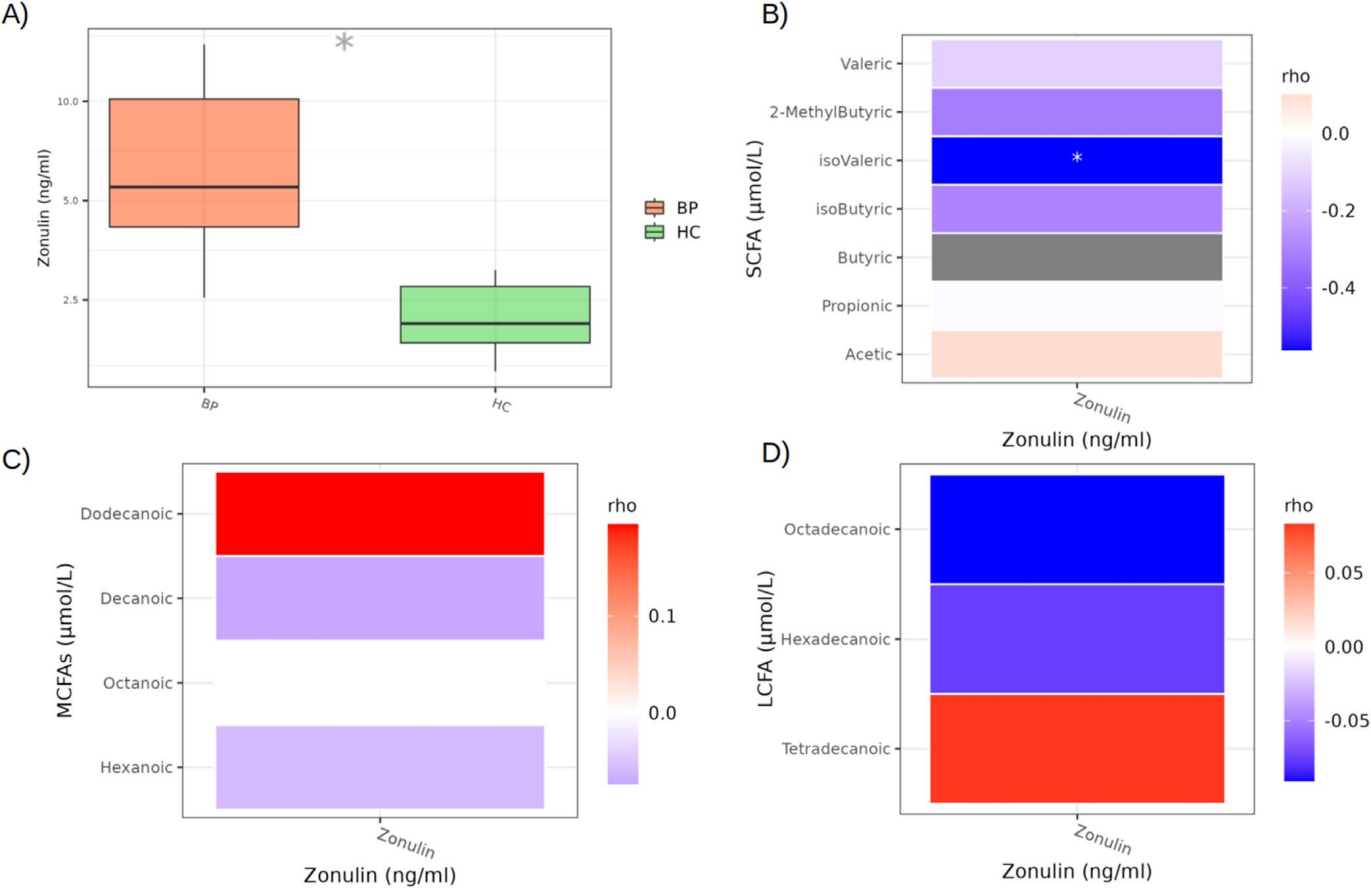
Boxplot representing the serum zonulin concentration (ng/mL) in HC and BP patients (A). Heatmaps of Spearman correlations between serum zonulin quantities and circulating SCFA (A), MCFA (B) and LCFA (C) concentrations (μmol/L) in BP patients. *p adjusted < 0.05. BP, bullous pemphigoid; HC, healthy control; LCFA, long‐chain fatty acid; MCFA, medium‐chain fatty acid; SCFA, short‐chain fatty acid.

To evaluate the intestinal permeability, serum zonulin concentrations (ng/mL) were measured by ELISA. Notably, BP patients showed significantly elevated zonulin levels (5.50 ± 6.0 ng/mL vs. 2.12 ± 0.89 ng/mL; *p* < 0.0001) compared to HC (Figure 2A). Furthermore, in BP patients, correlation analysis between zonulin and FFA abundances revealed an anti‐correlation with isovaleric acid (*ρ* = −0.560; *p*adj = 0.009) (Figure 2B). Conversely, no significant correlations were observed between zonulin and MCFAs (Figure 2C) and LCFAs (Figure 2D).

### 3.3 Correlation Among Circulating FFAs and Zonulin With Disease Activity

To gain insights into BP pathology, we analysed the correlations among FFAs, zonulin levels and clinical markers of disease activity, including the BPDAI score, BP180‐IgG and BP230‐IgG titers. However, no statistically significant associations were documented for these parameters (Table S2).

### 3.4 Comparison of FFA Profiles Among HC, BP and PV Patients

To assess differences in circulating FFAs among HC, patients with BP and PV patients, firstly we applied the Kruskal–Wallis test, which revealed significant group‐level differences in several FFAs (Table S3).

**FIGURE 3:**
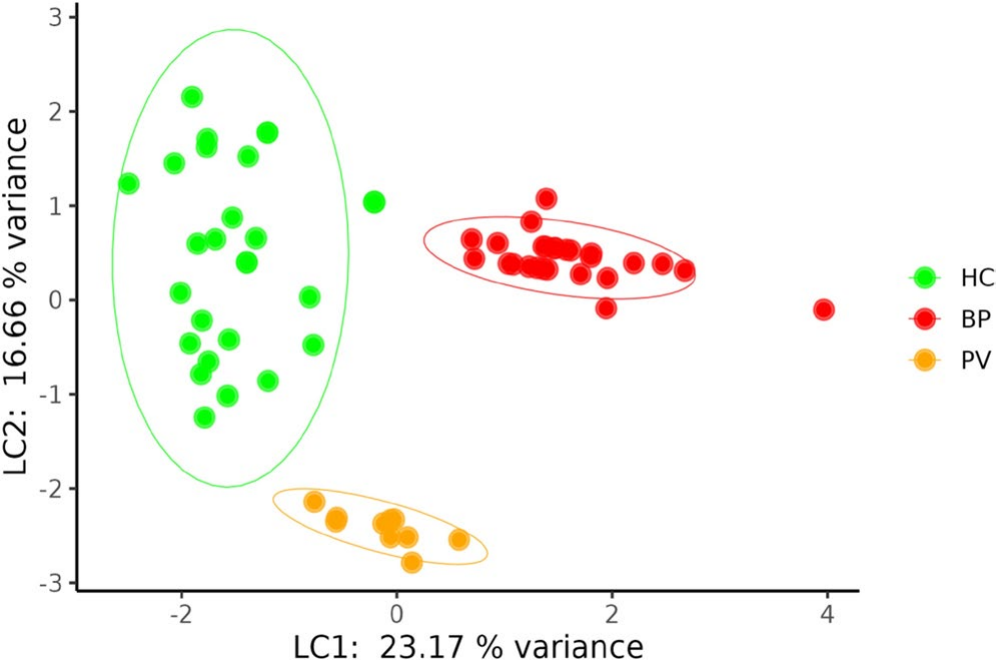
sPLS‐DA of the free fatty acids quantities shows a clear separation between BP, PV and HC. The ellipses display the 95% confidence interval for each group centroid. BP, bullous pemphigoid; HC, healthy control; LC1, latent component 1; LC2, latent component 2; PV, pemphigus vulgaris.

In detail, pairwise comparisons showed that SCFA profiles differed significantly between all groups (BP vs. PV: *p*adj = 0.0001; BP vs. HC: *p*adj = 0.0003; PV vs. HC: *p*adj = 0.0001). Similar profiles were observed for MCFAs (BP vs. PV: *p*adj = 0.0001; BP vs. HC: *p*adj = 0.0002; PV vs. HC: *p*adj = 0.001) and LCFAs (BP vs. PV: *p*adj = 0.0001; BP vs. HC: *p*adj = 0.0001; PV vs. HC: *p*adj = 0.001) (Figure S3). Additionally, analysis of total FFA amounts revealed that BP patients exhibited significantly lower SCFA levels compared to both PV (*p*adj = < 0.001) and HC (*p*adj = 9e‐4). In contrast, BP patients showed significantly higher MCFA abundances compared to both PV (*p*adj = 0.01) and HC (*p*adj = < 0.01). Regarding LCFAs, BP patients showed significantly higher levels than both PV (*p*adj < 0.0001) and HC (*p*adj = < 0.01). Moreover, a significant increase in LCFA levels was documented in PV patients compared to HC (*p*adj = 9e‐4) (Figure S3). To further identify the metabolites most strongly contributing to group separation among BP, PV and HC, we applied sPLS‐DA. This analysis successfully separated BP patients from HC along the first latent component (Figure 3), driven primarily by three key metabolites: propionic, octanoic and octadecanoic acids (Figure S4).

Subsequently, pairwise ROC curve analyses were conducted using the three key metabolites to evaluate their discriminative performance across all group comparisons (BP vs. HC, BP vs. PV and HC vs. PV). Specifically, propionic, octanoic and octadecanoic acids showed AUC values of 0.94, 0.93 and 1.00, respectively, showing excellent ability to distinguish BP patients from HC (Figure S5). In addition, the same metabolites effectively discriminated BP patients from PV patients, with AUC values of 0.87, 1.00 and 0.98, respectively (Figure S6). In the comparison between PV patients and HC, propionic, octanoic and octadecanoic acids demonstrated AUC values of 1.00, 0.79 and 0.98, respectively, confirming their strong discriminatory potential (Figure S7).

## 4 Discussion

Accumulating evidence suggests a strong connection between the GM and skin diseases [17]; however, the role of the GM in BP patients remains largely underexplored, especially in terms of functional profiling [11, 18]. Circulating FFAs have recently emerged as a critical pathway through which the GM remotely modulates systemic physiological and immune responses [19]. Of note, our previous studies have documented altered FFA profiles in various immune‐mediated diseases, including celiac disease [20], Crohn's disease [21], amyotrophic lateral sclerosis [22] and systemic sclerosis [23, 24]. In this context, our recent work identified a changed serum FFA profile in PV patients compared to HC, characterised by decreased propionic and butyric acids alongside elevated hexanoic and hexadecanoic acids, indicative of a shift toward a pro‐inflammatory condition [13].

In line with these findings, BP patients exhibited a distinct serum FFA signature compared to HC, further underscoring the potential involvement of GM‐derived metabolites in BP pathophysiology. Specifically, BP patients showed a significant decrease in total SCFAs, along with a statistically significant increase in both total MCFA and LCFA abundances compared to HC. In detail, among the panel of analysed SCFAs, the reduction of propionate is mainly noteworthy. Mechanistically, experimental studies have shown that propionate can attenuate T2 inflammation in allergic diseases [25, 26, 27], suggesting that its decreased availability may contribute to T2 inflammation in BP, although functional validation in humans is needed [28].

Regarding the fine analysis of each MCFA and LCFA, significantly elevated levels of octanoic, hexadecanoic and octadecanoic acids were found in BP patients compared to HC. MCFAs and LCFAs have been reported to promote inflammation by activating macrophages and dendritic cells and shifting immune responses toward a T helper (Th)1/Th17 phenotype [29]. While Th1 responses are not predominant in BP, Th17 cell activation has been implicated in chronic BP inflammation [30, 31], suggesting that modulation of Th17 pathways may have therapeutic potential [32]. Interestingly, decanoic acid, which was decreased in BP patients, has been shown to possess immunomodulatory effects, including inhibition of NF‐kB activation and stimulation of peroxisome proliferator‐activated receptor gamma (PPARγ), despite its generally pro‐inflammatory connotation [33, 34]. These observations highlight associations between specific FFAs and immune pathways, which require further functional validation in the context of BP.

Comparison of BP with another autoantibody‐mediated disease recently investigated by our group, such as systemic sclerosis, reveals both overlaps and differences in FFA alterations. For example, elevated octanoic acid levels were observed in both BP and SSc [23]. Although a significant reduction in SCFAs was observed in both conditions, BP was characterised by a selective decrease in propionate. Conversely, butyrate—the most extensively investigated SCFA—was markedly reduced in systemic sclerosis, while no significant changes were detected in BP [24], Collectively, these findings indicate that, while altered FFA metabolism may be common across immune‐mediated diseases, distinct FFA patterns occur, reflecting disease‐specific pathophysiology and immune‐regulatory differences.

Furthermore, we found that the serum zonulin, a biomarker of intestinal permeability, was significantly elevated in BP patients, marking the first report of this finding in BP. Zonulin release, often triggered by dysbiosis, disrupts gut barrier integrity by relaxing tight junctions, allowing bacterial and toxic metabolites to translocate into the systemic circulation. This ‘leaky gut’ condition may contribute to systemic immune activation and BP autoimmunity [35].

Additionally, building on our previously reported alterations in the FFAs profile of PV patients compared to HC—and given the shared pathogenetic mechanisms between PV and BP—we investigated potential group‐level differences in circulating FFAs to assess their role as disease biomarkers. In detail, pairwise comparisons revealed that BP patients exhibited significantly lower SCFA levels compared to both PV patients and HC. In contrast, both MCFA and LCFA abundances were significantly elevated in BP patients than in PV patients and HC. Notably, the marked decrease in SCFA levels of BP patients aligns with previous evidence indicating more severe GM dysregulation in BP compared to PV and HC [36]. This shift toward a pro‐inflammatory metabolic profile in BP is further supported by the increased levels of MCFAs and LCFAs, both of which are known to have pro‐inflammatory properties relative to the other groups. Finally, to identify the metabolites most strongly contributing to group separation among BP, PV and HC, we focused on propionic, octanoic and octadecanoic acids, which were identified by sPLS‐DA as key metabolites distinguishing BP patients from HC.

Notably, pairwise ROC curve analysis demonstrated the excellent discriminatory performance of these metabolites, with AUC values exceeding 0.9 in distinguishing BP patients from both PV patients and HC.

This study has some limitations, including its retrospective design and the lack of longitudinal data on FFA profiles following treatment. Furthermore, the associations between specific FFA alterations and the pathophysiology of BP remain speculative and warrant further experimental investigation. However, it provides the first evidence of altered FFA profiles and increased gut permeability in BP.

In conclusion, although further research is needed to clarify the precise FFAs role in BP pathogenesis, our findings highlight the strong diagnostic power of propionic, octanoic and octadecanoic acids. Moreover, the results emphasise the broader relevance of microbial‐derived metabolites in autoimmune diseases and underscore future dietary interventions aimed at modulating GM composition and SCFAs production as adjunctive strategies for BP patients undergoing immunosuppressive therapy.

## Author Contributions

Simone Baldi, Sara Bertorello and Roberto Maglie design the study; Simone Baldi, Sara Bertorello, Francesco Cei, Giulia Nannini, Elena Niccolai and Gianluca Bartolucci performed the experiments; Carlo Pipitò, Maria Efenesia Baffa, Emiliano Antiga and Roberto Maglie enrolled the patients and collected serum samples; Simone Baldi and Roberto Maglie wrote the manuscript; Emiliano Antiga and Amedeo amedei critically revised the manuscript; all the authors read and approved the final content of the manuscript.

## Conflicts of Interest

The authors declare no conflicts of interest.

## Supporting information


**Figure S1:** Boxplot representing total SCFAs (A), MCFAs (B) and LCFAs (C) amounts between HC and BP patients. Analyses were assessed using the Mann–Whitney test and asterisks (*) represent adj. *p* values < 0.05.
**Figure S2:** Principal Coordinate Analysis (PCoA) based on Bray–Curtis dissimilarity revealed significant differences in SCFAs (A), MCFAs (B) and LCFAs (C) profiles among BP, PV and HC groups. Statistical significance was assessed using PERMANOVA (9999 permutations), adjusting for sex and age as potential confounding variables.
**Figure S3:** Boxplots showing total SCFAs (A), MCFAs (B) and LCFAs (C) amounts across HC, PV patients and BP patients. Statistical comparisons were performed using the Kruskal–Wallis test, followed by post hoc Dunn's test. Asterisks indicate adjusted *p*‐values: * < 0.05, ** < 0.01, *** < 0.001.
**Figure S4:** Barplot of the loadings of FFAs selected on component 1 of the sPLS‐DA analysis. The values represent the contribution of each selected FFA to the group separation along this component. The three highlighted FFAs (propionic, octanoic and octadecanoic acids) showed the highest absolute loading values and were chosen for further analysis. The red dashed line represents an empirical threshold at 50% of the most negative loading value, used to guide feature selection.
**Figure S5:** ROC curves for the discrimination between BP patients and HC based on the concentrations of propionic (A), octanoic (B) and octadecanoic (C) acids. Panels (D), (E) and (F) show the corresponding optimal cutoff values identified for each metabolite, along with their confusion matrices illustrating classification performance.
**Figure S6:** ROC curves for the discrimination between BP and PV patients and HC based on the concentrations of propionic (A), octanoic (B) and octadecanoic (C) acids. Panels (D), (E) and (F) show the corresponding optimal cutoff values identified for each metabolite, along with their confusion matrices illustrating classification performance.
**Figure S7:** ROC curves for the discrimination between PV patients and HC based on the concentrations of propionic (A), octanoic (B) and octadecanoic (C) acids. Panels (D), (E) and (F) show the corresponding optimal cutoff values identified for each metabolite, along with their confusion matrices illustrating classification performance.
**Table S1:** Serum FFAs abundances (μmol/L) of HC and BP patients. Data are presented as median (interquartile range, IQR). adj. *p* values (Benjamini–Hochberg correction) were calculated using the Mann–Whitney test and were considered statistically significant if < 0.05.
**Table S2:** Results of Spearman correlations between FFAs and clinical parameters, including the BPDAI score, BP180‐IgG and BP230IgG titres. *p*‐values were adjusted with the Benjamini–Hochberg correction, with *p* values less than 0.05 considered statistically significant.
**Table S3:** Results of the Kruskal–Wallis test evaluating differences in FFA levels among BP patients, PV patients and HC. To identify specific group differences, Dunn's post hoc test was performed. Reported *p*‐values have been adjusted for multiple comparisons using the Benjamini–Hochberg correction method.

## Data Availability

The data that support the findings of this study are available from the corresponding author upon reasonable request.
