# Supplementary material for: A Specific Signature of Circulating Free Fatty Acid Discriminates Bullous Pemphigoid From Pemphigus Vulgaris and Healthy Controls
Source: Exp Dermatol. 2026 Feb 20;35(2):e70228. doi: 10.1111/exd.70228 (PMC12921840; doi:10.1111/exd.70228)
Supplement: Supplementary file 1 — Figure S1: Boxplot representing total SCFAs (A), MCFAs (B) and LCFAs (C) amounts between HC and BP patients. Analyses were assessed using the Mann–Whitney test and asterisks (*) represent adj. p values < 0.05. Figure S2: Principal Coordinate Analysis (PCoA) based on Bray–Curtis dissimilarity revealed significant differences in SCFAs (A), MCFAs (B) and LCFAs (C) profiles among BP, PV and HC groups. Statistical significance was assessed using PERMANOVA (9999 permutations), adjusting for sex and age as potential confounding variables. Figure S3: Boxplots showing total SCFAs (A), MCFAs (B) and LCFAs (C) amounts across HC, PV patients and BP patients. Statistical comparisons were performed using the Kruskal–Wallis test, followed by post hoc Dunn's test. Asterisks indicate adjusted p‐values: * < 0.05, ** < 0.01, *** < 0.001. Figure S4: Barplot of the loadings of FFAs selected on component 1 of the sPLS‐DA analysis. The values represent the contribution of each selected FFA to the group separation along this component. The three highlighted FFAs (propionic, octanoic and octadecanoic acids) showed the highest absolute loading values and were chosen for further analysis. The red dashed line represents an empirical threshold at 50% of the most negative loading value, used to guide feature selection. Figure S5: ROC curves for the discrimination between BP patients and HC based on the concentrations of propionic (A), octanoic (B) and octadecanoic (C) acids. Panels (D), (E) and (F) show the corresponding optimal cutoff values identified for each metabolite, along with their confusion matrices illustrating classification performance. Figure S6: ROC curves for the discrimination between BP and PV patients and HC based on the concentrations of propionic (A), octanoic (B) and octadecanoic (C) acids. Panels (D), (E) and (F) show the corresponding optimal cutoff values identified for each metabolite, along with their confusion matrices illustrating classification performance. Figure S [file EXD-35-e70228-s001.docx]

**SUPPLEMENTARY FIGURES**


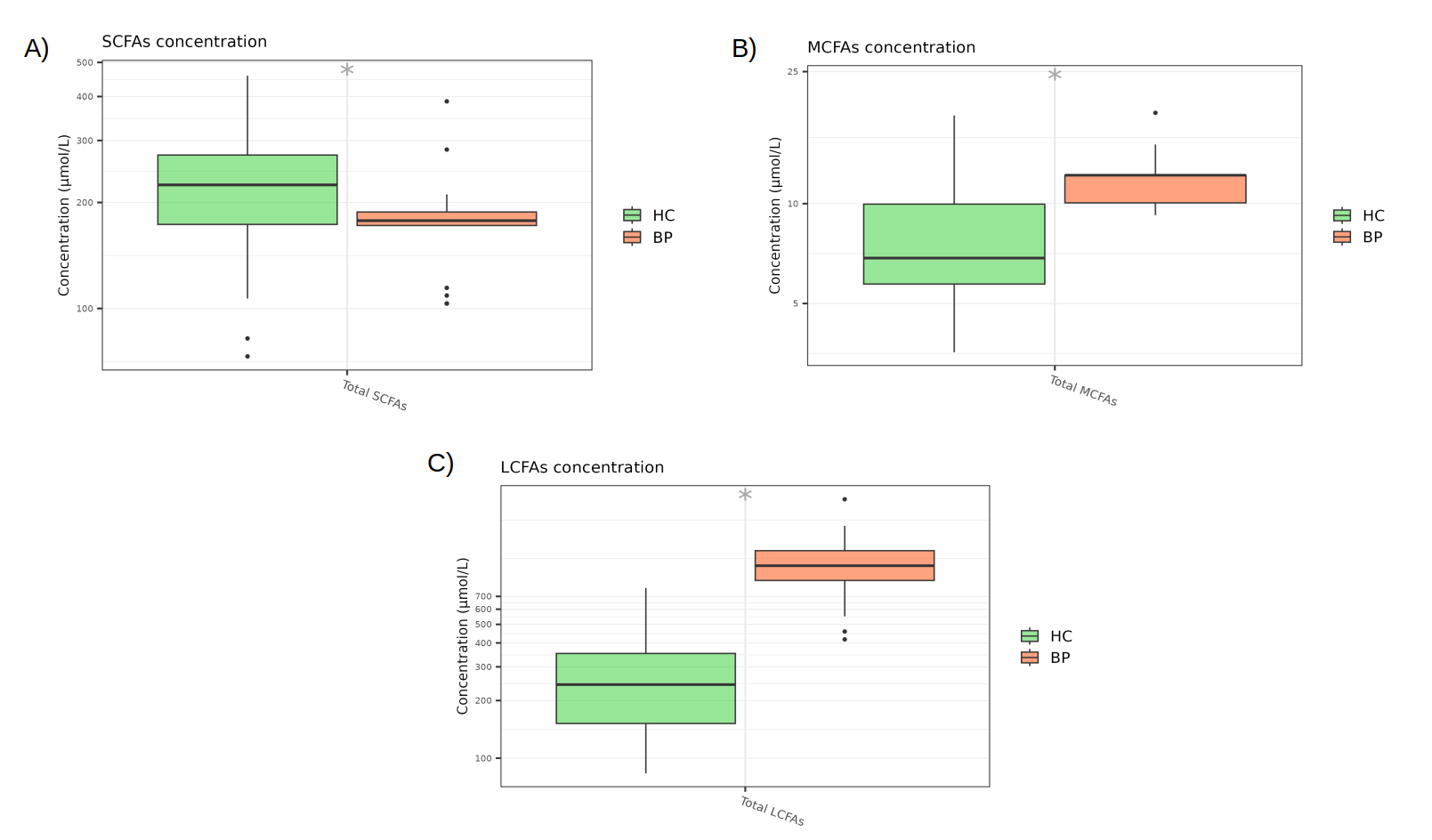


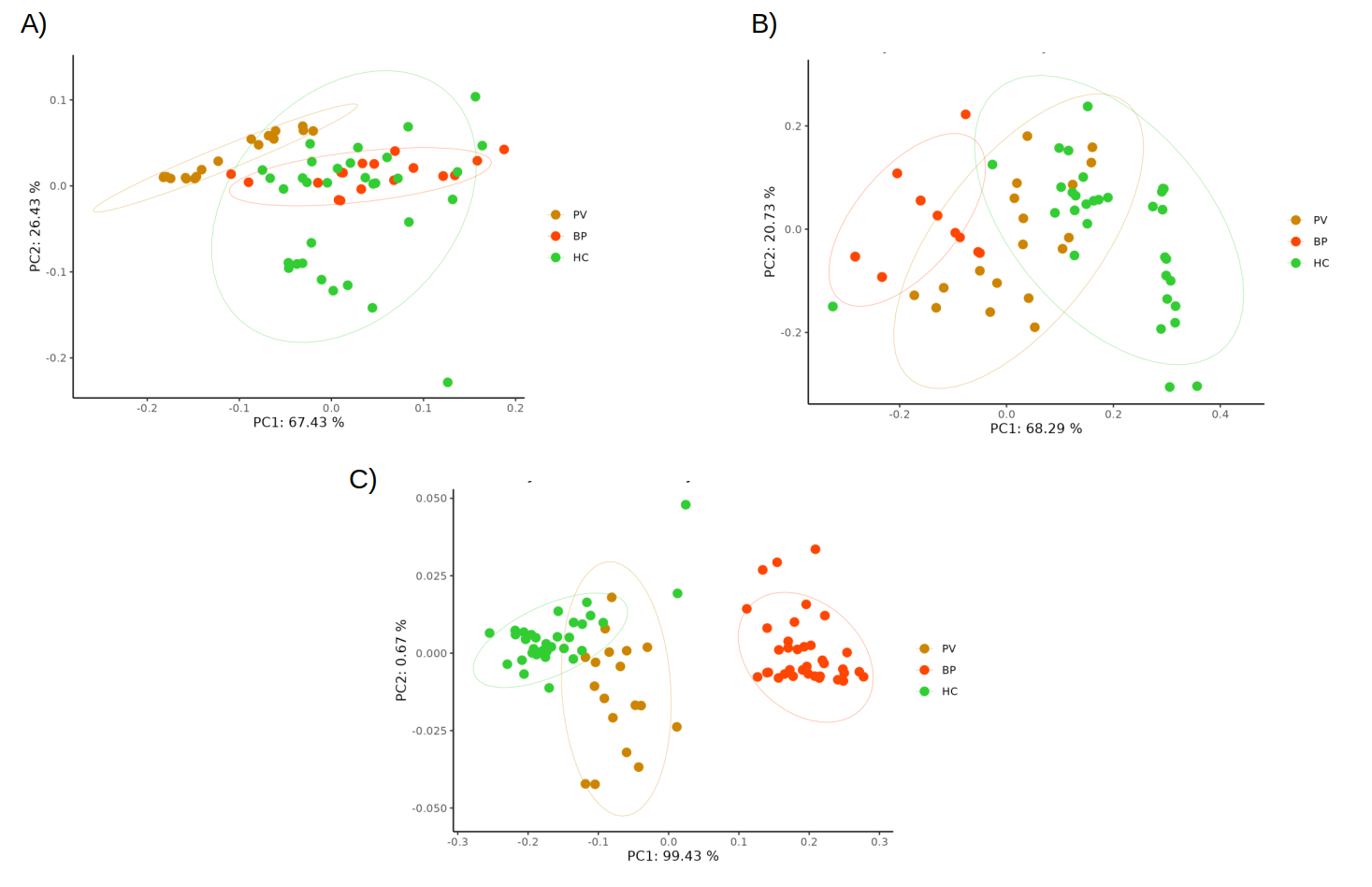
**Figure S1.** Boxplot representing total SCFAs (A), MCFAs (B) and LCFAs (C) amounts between HC and BP patients. Analyses were assessed using the Mann-Whitney test and asterisks (*) represent adj.p values <0.05.

**Figure S2.** Principal Coordinate Analysis (PCoA) based on Bray-Curtis dissimilarity revealed significant differences in SCFAs (A), MCFAs (B) and LCFAs (C) profiles among BP, PV, and HC groups. Statistical significance was assessed using PERMANOVA (9,999 permutations), adjusting for sex and age as potential confounding variables.

**
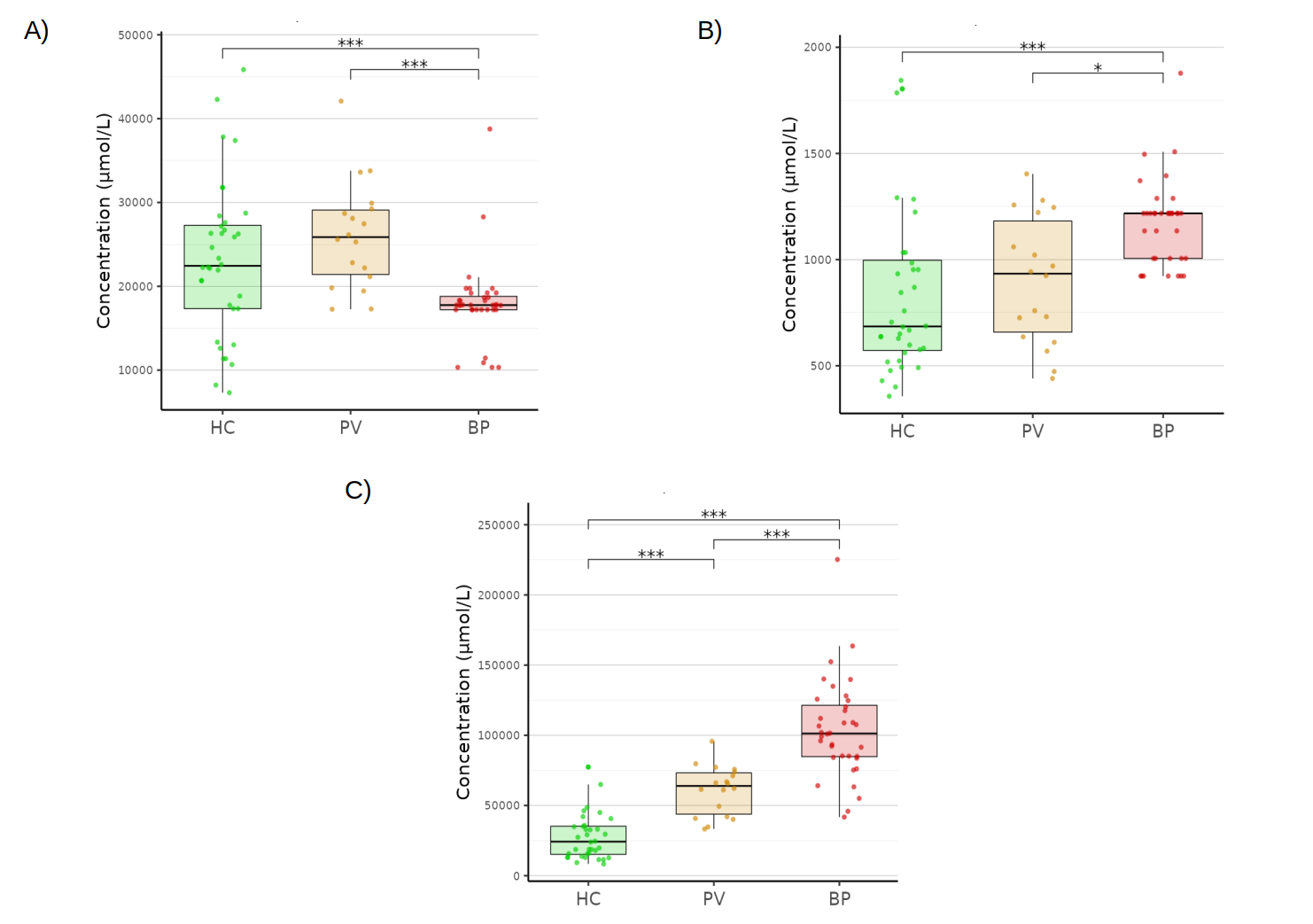
**

**Figure S3.** Boxplots showing total SCFAs (A), MCFAs (B) and LCFAs (C) amounts across HC, PV patients, and BP patients. Statistical comparisons were performed using the Kruskal–Wallis test, followed by post hoc Dunn’s test. Asterisks indicate adjusted p-values: * < 0.05, ** < 0.01, *** < 0.001**.**


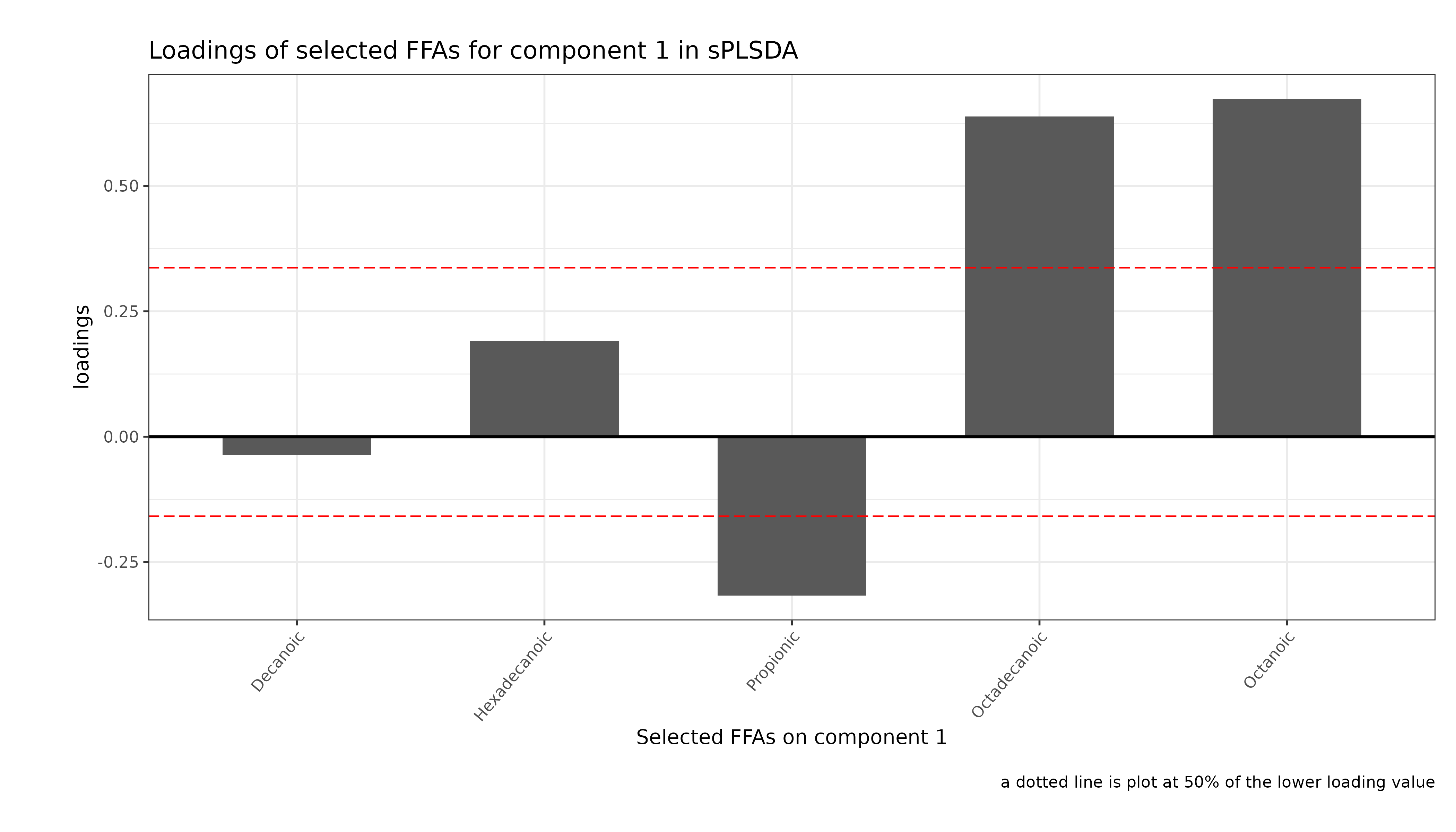


**Figure S4.** Barplot of the loadings of FFAs selected on component 1 of the sPLS-DA analysis. The values represent the contribution of each selected FFA to the group separation along this component. The three highlighted FFAs (propionic, octanoic and octadecanoic acids) showed the highest absolute loading values and were chosen for further analysis. The red dashed line represents an empirical threshold at 50% of the most negative loading value, used to guide feature selection.


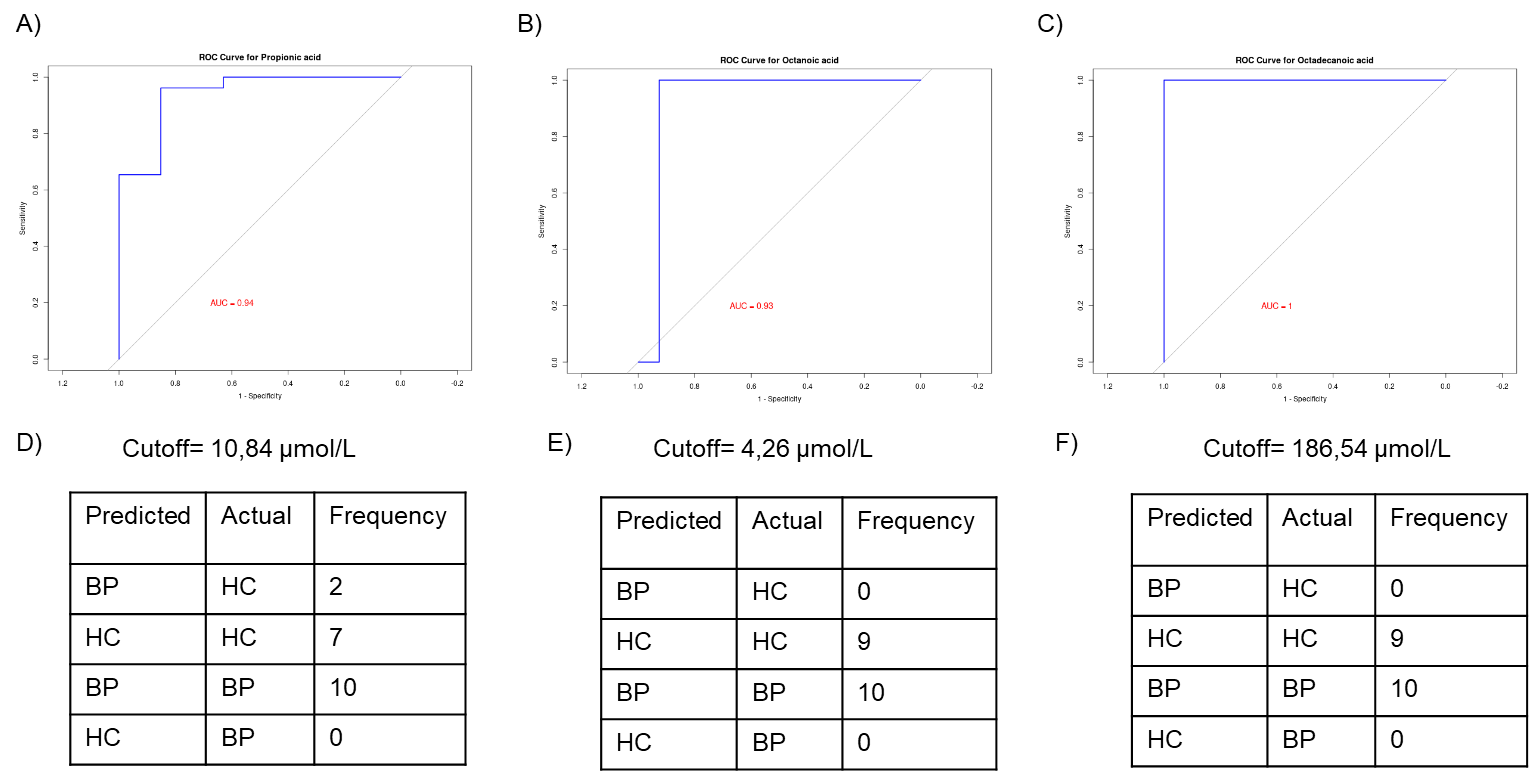


**Figure S5.** ROC curves for the discrimination between BP patients and HC based on the concentrations of propionic (A), octanoic (B), and octadecanoic (C) acids. Panels (D), (E), and (F) show the corresponding optimal cutoff values identified for each metabolite, along with their confusion matrices illustrating classification performance.


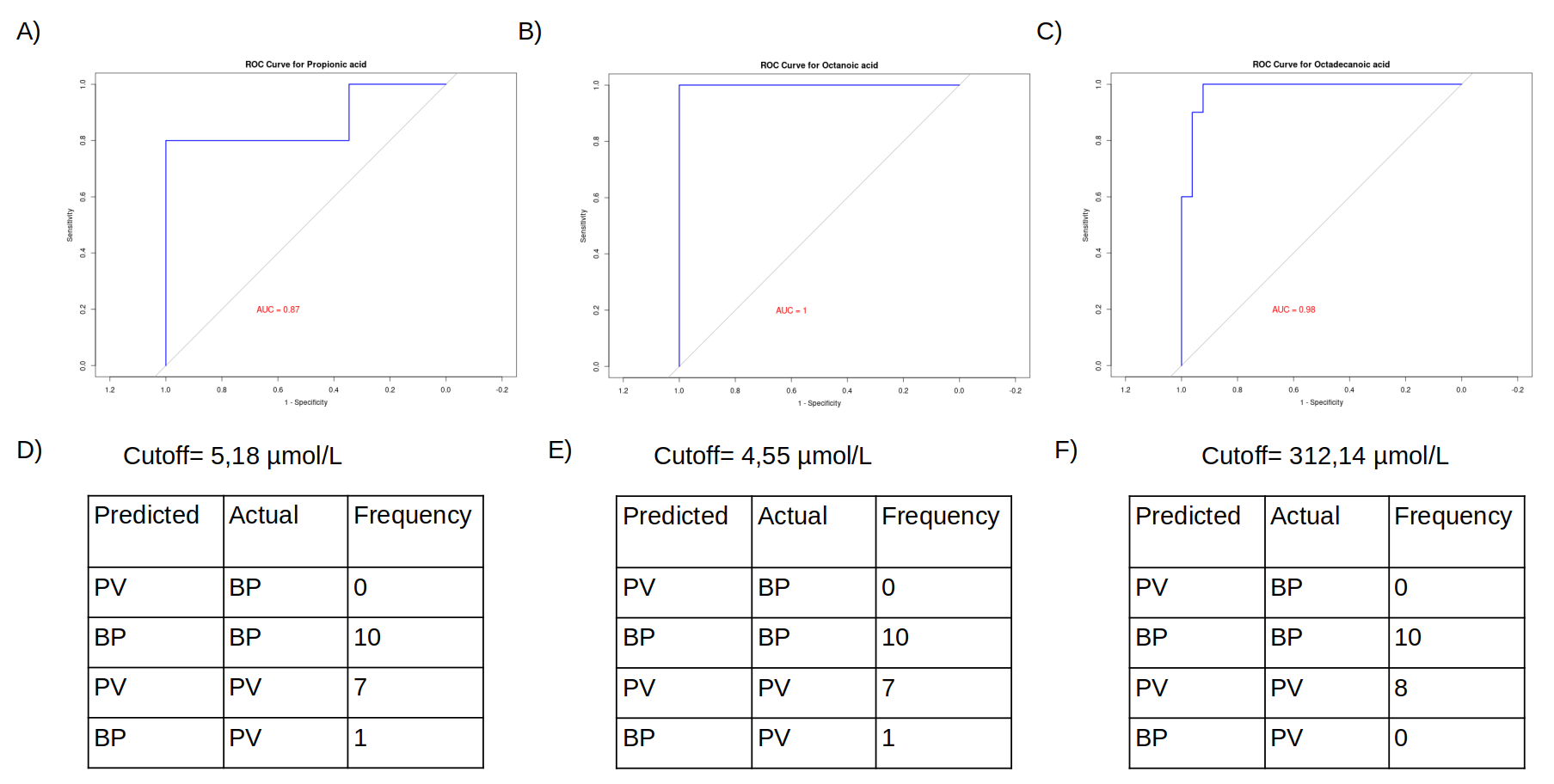


**Figure S6.** ROC curves for the discrimination between BP and PV patients and HC based on the concentrations of propionic (A), octanoic (B), and octadecanoic (C) acids. Panels (D), (E), and (F) show the corresponding optimal cutoff values identified for each metabolite, along with their confusion matrices illustrating classification performance.


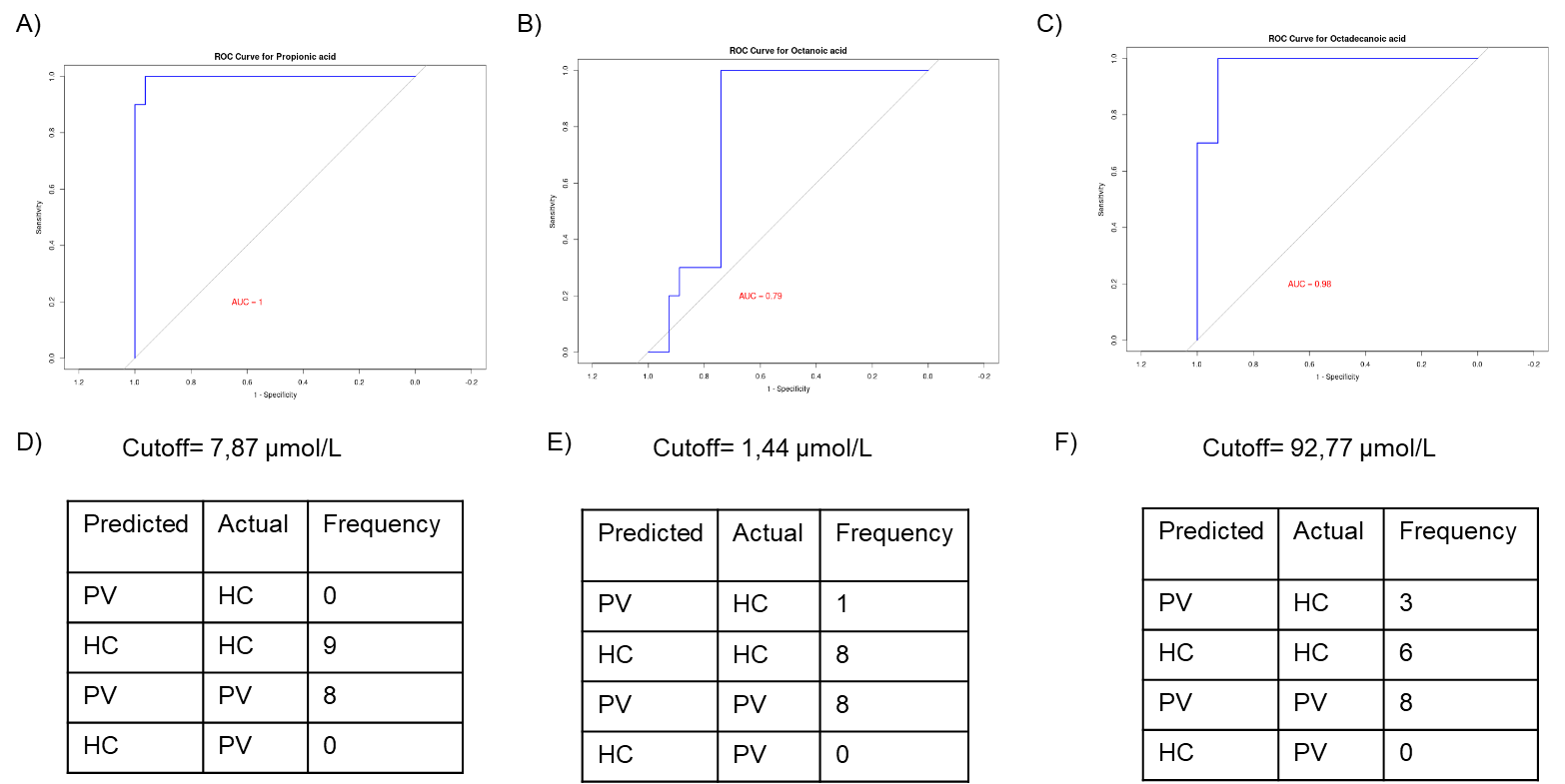


**Figure S7.** ROC curves for the discrimination between PV patients and HC based on the concentrations of propionic (A), octanoic (B), and octadecanoic (C) acids. Panels (D), (E), and (F) show the corresponding optimal cutoff values identified for each metabolite, along with their confusion matrices illustrating classification performance.

**SUPPLEMENTARY TABLES**

**Table S1.** Serum FFAs abundances (µmol/L) of HC and BP patients. Data are presented as median (interquartile range, IQR). adj. p values (Benjamini–Hochberg correction) were calculated using the Mann–Whitney test and were considered statistically significant if <0.05.

| **FFA (µmol/L)** | **BP** | **HC** | **p. adj** |
| --- | --- | --- | --- |
| Total SCFAs | 177.62 (15.91) | 224.42 (99.48) | 0.002 |
| Acetic acid | 137.54 (0.00) | 168.13 (74.47) | 7.25e-4 |
| Propionic acid | 5.22 (5.22) | 17.64 (8.83) | 2.95e-10 |
| Butyric acid | 8.47 (0.00) | 5.85 (3.42) | 6.35e-08 |
| isoButyric acid | 9.07 (2.26) | 12.39 (12.91) | 0.794 |
| 2-Methylbutyric acid | 5.57 (0.00) | 8.14 (10.54) | 0.999 |
| isoValeric acid | 11.08 (5.54) | 13.19 (17.40) | 0.207 |
| Valeric acid | 0.68 (0.00) | 0.59 (0.49) | 0.009 |
| Total MCFAs | 12.18 (2.12) | 6.85 (4.24) | 3.39e-05 |
| Hexanoic acid | 1.66 (0.82) | 1.55 (1.53) | 0.189 |
| Octanoic | 5.57 (0.00) | 1.09 (1.01) | 1.42e-11 |
| Decanoic | 0.70 (0.00) | 1.65 (0.36) | 1.08e-08 |
| Dodecanoic | 4.25 (2.12) | 2.51 (2.27) | 0.031 |
| Total LCFAs | 1012.59 (365.58) | 242.32 (200.46) | 2.37e-12 |
| Tetradecanoic | 9.64 (5.69) | 12.63 (13.58) | 0.311 |
| Hexadecanoic | 420.16 (154.08) | 178.80 (158.50) | 1.06e-7 |
| Octadecanoic | 603.11 (272.38) | 47.66 (32.04) | 9.12e-13 |

**Table S2.** Results of Spearman correlations between FFAs and clinical parameters, including the BPDAI score, BP180-IgG and BP230IgG titres. P-values were adjusted with the Benjamini–Hochberg correction, with p values less than 0.05 considered statistically significant.

| **Correlation** | **ρ** | **p. adj** |
| --- | --- | --- |
| BPDAI vs Acetic acid | -0.254 | 0.404 |
| BPDAI vs Propionic acid | -0.084 | 0.699 |
| BPDAI vs Butyric acid | -0.087 | 0.699 |
| BPDAI vs isoButyric acid | -0.098 | 0.699 |
| BPDAI vs 2-Methylbutyric acid | -0.066 | 0.699 |
| BPDAI vs isoValeric acid | -0.090 | 0.699 |
| BPDAI vs Valeric acid | 0.385 | 0.121 |
| BPDAI vs Hexanoic acid | -0.026 | 0.876 |
| BPDAI vs Octanoic acid | -0.039 | 0.876 |
| BPDAI vs Decanoic acid | -0.062 | 0.876 |
| BPDAI vs Dodecanoic acid | -0.222 | 0.577 |
| BPDAI vs Tetradecanoic acid | -0.089 | 0.893 |
| BPDAI vs Hexadecanoic acid | 0.055 | 0.893 |
| BPDAI vs Octadecanoic acid | -0.023 | 0.893 |
| BP230 vs Acetic acid | -0.293 | 0.513 |
| BP230 vs Propionic acid | -0.353 | 0.513 |
| BP230 vs Butyric acid | -0.256 | 0.612 |
| BP230 vs isoButyric acid | -0.151 | 0.604 |
| BP230 vs 2-Methylbutyric acid | -0.365 | 0.532 |
| BP230 vs isoValeric acid | -0.237 | 0.516 |
| BP230 vs Valeric acid | -0.411 | 0.513 |
| BP230 vs Hexanoic acid | 0.159 | 0.586 |
| BP230 vs Octanoic acid | 0.384 | 0.380 |
| BP230 vs Decanoic acid | 0.327 | 0.380 |
| BP230 vs Dodecanoic acid | 0.395 | 0.380 |
| BPDAI vs Tetradecanoic acid | 0.207 | 0.476 |
| BPDAI vs Hexadecanoic acid | 0.345 | 0.340 |
| BPDAI vs Octadecanoic acid | 0.432 | 0.340 |
| BP180 vs Acetic acid | 0.389 | 0.513 |
| BP180 vs Propionic acid | -0.293 | 0.513 |
| BP180 vs Butyric acid | -0.353 | 0.623 |
| BP180 vs isoButyric acid | -0.211 | 0.604 |
| BP180 vs 2-Methylbutyric acid | -0.284 | 0.574 |
| BP180 vs isoValeric acid | -0.237 | 0.516 |
| BP180 vs Valeric acid | 0.298 | 0.513 |
| BP180 vs Hexanoic acid | 0.159 | 0.586 |
| BP180 vs Octanoic acid | 0.297 | 0.380 |
| BP180 vs Decanoic acid | 0.327 | 0.380 |
| BP180 vs Dodecanoic acid | 0.395 | 0.380 |
| BP180 vs Tetradecanoic acid | 0.207 | 0.476 |
| BP180 vs Hexadecanoic acid | 0.345 | 0.340 |
| BP180 vs Octadecanoic acid | 0.432 | 0.340 |

**Table S3.** Results of the Kruskal–Wallis test evaluating differences in FFA levels among BP patients, PV patients and HC. To identify specific group differences, Dunn’s post hoc test was performed. Reported p-values have been adjusted for multiple comparisons using the Benjamini–Hochberg correction method.

| **FFA (µmol/L)** | **Kruskal-Wallis p-value** | **Comparison** | **Dunn adj. p-value** |
| --- | --- | --- | --- |
| Total SCFAs | 0.0023 | HC vs BP | 0.0009 |
|  |  | HC vs PV | 0.0533 |
|  |  | BP vs PV | 0.001 |
| Acetic acid | 3.09e-07 | HC vs BP | 0.0009 |
|  |  | HC vs PV | 2.48e-08 |
|  |  | BP vs PV | 0.0030 |
| Propionic acid | 7.26e-14 | HC vs BP | 3.83e-08 |
|  |  | HC vs PV | 8.62e-14 |
|  |  | BP vs PV | 0.0022 |
| Butyric acid | 7.85e-14 | HC vs BP | 1.22e-05 |
|  |  | HC vs PV | 1.66e-05 |
|  |  | BP vs PV | 4.94e-05 |
| isoButyric acid | 0.0420 | HC vs BP | 0.6472 |
|  |  | HC vs PV | 0.0572 |
|  |  | BP vs PV | 0.0432 |
| 2-Methylbutyric acid | 0.8549 | HC vs BP | 1 |
|  |  | HC vs PV | 0.8047 |
|  |  | BP vs PV | 1 |
| isoValeric acid | 0.2983 | HC vs BP | 0.2903 |
|  |  | HC vs PV | 0.5704 |
|  |  | BP vs PV | 0.8036 |
| Valeric acid | 4.52e-08 | HC vs BP | 0.0072 |
|  |  | HC vs PV | 9.32e-05 |
|  |  | BP vs PV | 1.72e-09 |
| Total MCFAs | 3.38e-04 | HC vs BP | 0.001 |
|  |  | HC vs PV | 0.1524 |
|  |  | BP vs PV | 0.0132 |
| Hexanoic acid | 0.0002 | HC vs BP | 0.2087 |
|  |  | HC vs PV | 0.0001 |
|  |  | BP vs PV | 0.0037 |
| Octanoic | 1.24e-13 | HC vs BP | 6.91e-15 |
|  |  | HC vs PV | 0.027 |
|  |  | BP vs PV | 3.10e-05 |
| Decanoic | 1.75e-06 | HC vs BP | 4.37e-07 |
|  |  | HC vs PV | 0.6549 |
|  |  | BP vs PV | 0.0001 |
| Dodecanoic | 0.042 | HC vs BP | 0.053 |
|  |  | HC vs PV | 0.6865 |
|  |  | BP vs PV | 0.1029 |
| Total LCFAs | 2.33e-11 | HC vs BP | 0.001 |
|  |  | HC vs PV | 0.0009 |
|  |  | BP vs PV | 0.0007 |
| Tetradecanoic | 0.4834 | HC vs BP | 0.7423 |
|  |  | HC vs PV | 0.8717 |
|  |  | BP vs PV | 0.6504 |
| Hexadecanoic | 3,17e-07 | HC vs BP | 1.55e-07 |
|  |  | HC vs PV | 3.35e-05 |
|  |  | BP vs PV | 0.8365 |
| Octadecanoic | 5.12e-16 | HC vs BP | 1.39e-17 |
|  |  | HC vs PV | 0.0004 |
|  |  | BP vs PV | 0.0005 |
